# Evaluation of periodontitis parameters and plaque examination by microscopy: a report on 20 patients

**DOI:** 10.3389/fdmed.2024.1451698

**Published:** 2024-11-21

**Authors:** Mark Bonner

**Affiliations:** Dental Clinic Limited to Gum Disease, Victoriaville, QC, Canada

**Keywords:** periodontitis, *Entamoeba gingivalis*, periodontal treatment, oral biofilm, phase contrast microscopy, biological markers

## Abstract

The protozoan parasite *Entamoeba gingivalis* has long been detected in diseased gingival pockets. The parasite is found in 81% of diseased sites using PCR and in up to 100% using microscopy, whereas it is mostly absent from healthy gingival sites. The purpose of this study on 20 periodontitis patients was to analyze the characteristic biofilm using phase-contrast microscopy and evaluate the results of a novel antiparasitic, anti-inflammatory therapeutical approach. The therapeutic strategy, termed “Periodontal Healing Protocol Bonner Dunoyé” (PHPBD), is implemented in monthly appointments for 8 months, and a control visit at one year. It involves a disinfection protocol, subgingival calculus removal, patient training and the microscopic analysis of periodontal biofilm sulci. The practitioner also records bleeding on probing (BOP) and pocket depth (PD) to quantify healing. In all cases, the initial biofilm composed mainly of parasites, neutrophils, spirochetes, and other motile bacteria was progressively replaced by a white blood cell-free biofilm, consisting of motionless coccoid bacteria, filaments, and epithelial cells, indicative of healthy periodontium. Results were stable from month 8 to month 12. At one year, both BOP and PD values were greatly reduced (96%–100% decrease) compared to initial levels. The average sulcus clinical pocket healing toward the 1–3 mm PD group teeth was close to 99% overall patients. In conclusion, implementation of PHPBD appears to result in complete healing of periodontitis within 12 months, as determined by BOP, PD, biofilm microscopic monitoring and elimination of motile bacteria, parasites, and inflammatory cells. Thus, periodontal dysbiosis can be microscopically guided toward predictable eubiosis. Further studies are needed to evaluate long-term benefits.

## 1 Introduction

The relevance of oral bacteria in human health and disease is a well-investigated though growing field of research. However, the study of parasites in the digestive tract has been mainly limited to pathogenic intestinal ones. Interestingly, *Entamoeba gingivalis* (*E. gingivalis*), which is found in the mouth, was the first endosymbiotic amoeba discovered in humans, in 1849 (1) and was associated with gingival inflammation and pyorrhea alveolaris as early as 1914 (2, 3). Transmission of the parasite is still under debate—though it was hypothesized that it may be spread by saliva droplets, by direct contact or by sharing tools (4)—as is the existence of a resistant form, the cyst, identified in most other Entamoebidae of medical importance.

In our previous studies, we have shown that *E. gingivalis* was unequivocally identified by polymerase chain reaction (PCR) using specific genetic markers in patients with periodontitis, while being mostly absent from healthy sites (5). This correlation between parasite identification and disease was even stronger using microscopy as seen in a cohort of 632 patients (6).

Together with the phylogenetic vicinity with the pathogenic intestinal species *Entamoeba histolytica* (*E. histolytica)*, these observations suggested an etiological role for *E. gingivalis* in periodontitis. The therapeutic benefits observed following administration of the anti-parasitic drug emetine hydrochloride to periodontitis patients reinforced this hypothesis (7). Similarly, recent therapeutic strategies targeting parasites allowed periodontitis resolution and bone regeneration, linking cure to evolution of the crevicular biofilm (8–11). *Trichomonas tenax* (*T. tenax)* although less frequently present, is similarly associated with severe periodontal disease (12). Interestingly, this parasite was shown to cause damage to different mammalian cells *in vitro* (13).

Recent advances in the field of periodontitis have demonstrated that important pathogens other than bacteria can participate in modulating the sulcus environment and consequently cause dysbiosis, supporting an important role in the pathophysiology of periodontitis (14, 15). *E. gingivalis*, like other parasitic protozoa, uses trogocytosis to ingest material from target blood cells (16). The parasite induces a strong immune response, particularly the expression of TNF-α, IL8 and proinflammatory chemokines, hence contributing to impair barrier integrity (15). It has been suggested that elimination of *E. gingivalis* from the inflamed periodontal pockets by an antiparasitic therapy might have the potential to arrest and resolve oral inflammation and improve periodontal healing (15).

In addition to published data, our many years of clinical experience in periodontal microscopy have taught us that adequate hygiene is not sufficient to eliminate mouth protozoa in most cases of periodontitis. We have proposed a normalized protocol, which we named PHPBD and deposited at the American Academy of Periodontology library in 2015. As mentioned above, a previous study suggested that this protocol could lead to a cure (6). It was previously determined that sampling on a single site to evaluate periodontitis evolution can be misleading (17). In this study, for which 20 patients were treated normally, we measured all teeth and performed analysis of BOP and PD. Concomitantly, the biofilm was analyzed microscopically to monitor microbes and inflammatory cells as part of the regular treatment.

## 2 Materials and methods

### 2.1 Patient recruitment

Data for this study originate from 20 patients concerned with their periodontal care who visited our periodontal practice clinic in Victoriaville, Québec, Canada. Out of a total of 162 patients seen for gum examination, 13 were considered healthy, whereas 58 received a diagnosis of gum disease. Eight were diagnosed gingivitis, 50 diagnosed periodontitis of Stage I–II–III–IV (18, 11, 20 and 1, respectively). The remainder were not considered for this study as they chose to refuse treatment (15), were on recall program (9), were mentally impaired (1), or presented with disqualifying conditions such as a need for emergency periodontal condition or grafting procedure (66). The results presented here are from the 20 Stage III patients, who all opted to undergo non-surgical treatment. All patients signed a consent form after having considered all the different treatment options proposed. Fourteen were females and six were males; age ranged from 26 to 70 years old.

### 2.2 Recommendations to patients

Patients were asked not to brush nor floss for 12 h prior to every appointment to help standardize subgingival biofilm analyses. Oral hygiene was done at the clinic after every plaque examination, with the purpose to teach patients adequate and complete mouth cleaning and disinfection techniques. Every appointment lasted 50–60 min, divided in three parts of about equal duration: biology education for therapy, clinical periodontology, and patient autonomy regarding hygiene and disinfection methods. Eight appointments were given once every month, followed by a last appointment at 1 year after initiation of the treatment and a second supportive recall three months later if needed. During therapy, 4 questionnaires were conducted, allowing the patient to understand the suspected pathophysiology and transmission of the disease, emphasizing on anamnesis, parasitology contamination, tropical visit area, and personal experiences.

### 2.3 Evaluation of periodontitis parameters

During the first and last appointments, BOP was assessed using 6 sites per tooth on every tooth: all teeth with at least one point bleeding on probing were considered positive. Scores are presented as the total number of positive teeth. PD was measured by probing on 6 sites per tooth on every tooth. For each category, the corresponding number of positive sites were summed. Microscopic screening was done at every appointment on fresh smears of crevicular biofilm taken from the 3 deepest affected periodontal pockets each from a different tooth. We observed those deep sulci plaque specimens by phase-contrast microscopy at 100× magnification (scanning) and 1,000× (spotting), mounted on patient saliva to avoid deformation of protozoa from water or saline. A hospital-grade phase contrast microscope was used at every appointment for all microscopy analyses. Halitosis was evaluated by patients themselves after flossing 2 deep area and smelling with their own sense on a 1–5 basis.

### 2.4 Therapy

**Figure 1:**
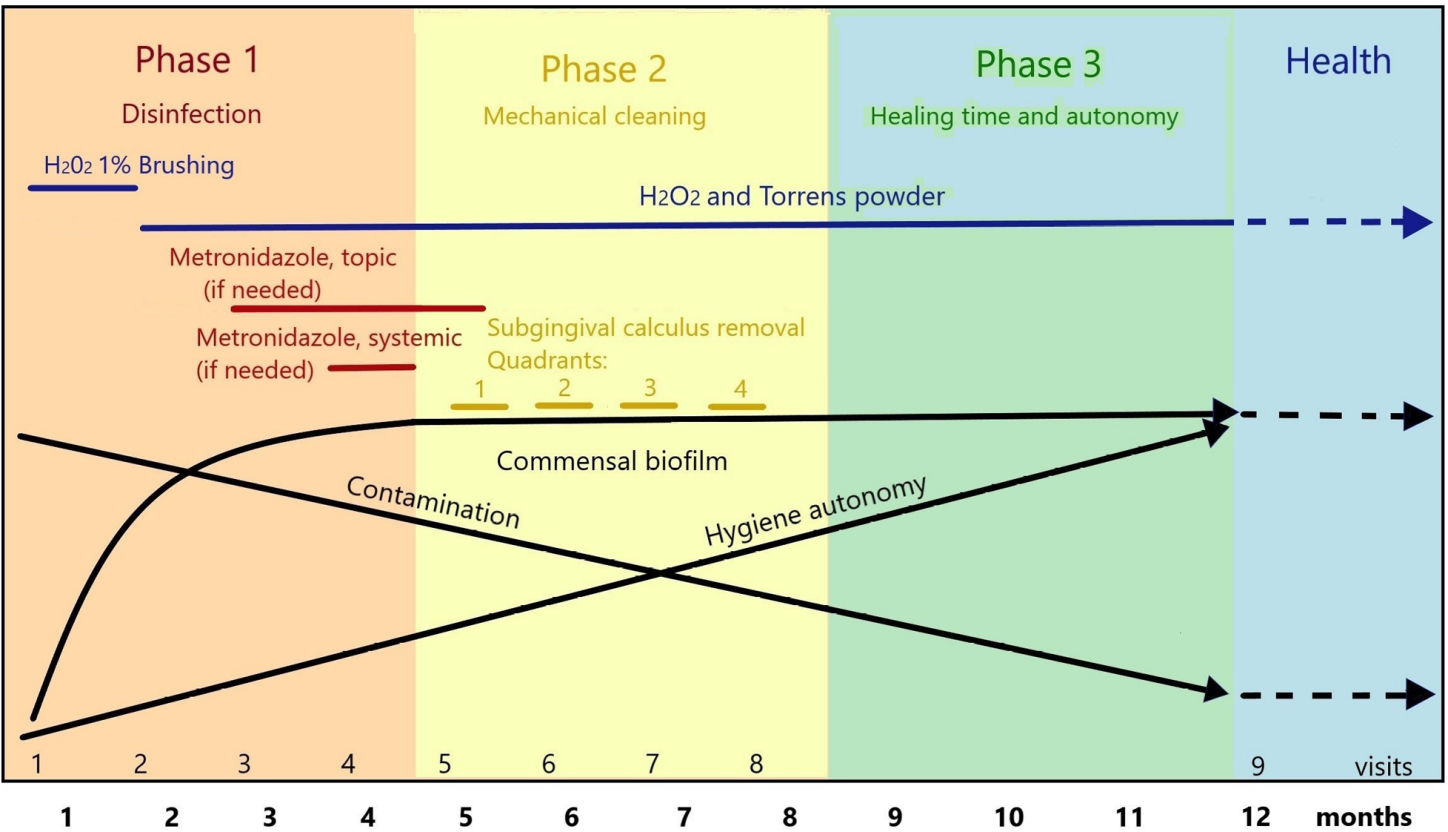
Rationale for anti-parasitic treatment and biofilm management in 1-year periodontal therapy following the PHPBD protocol. The first 4 months are mostly dedicated to disinfection confirmed by microscopy. Months 5–8 are dedicated to gentle calculus removal in an obliged commensal microbiota. The last 4 months allow time to complete healing. During this process, patient is given training in hygiene and disinfection techniques within the office, is educated toward avoiding primary gingivitis and parasitic transmission and contamination from social interactions and environment.

**Figure 2:**
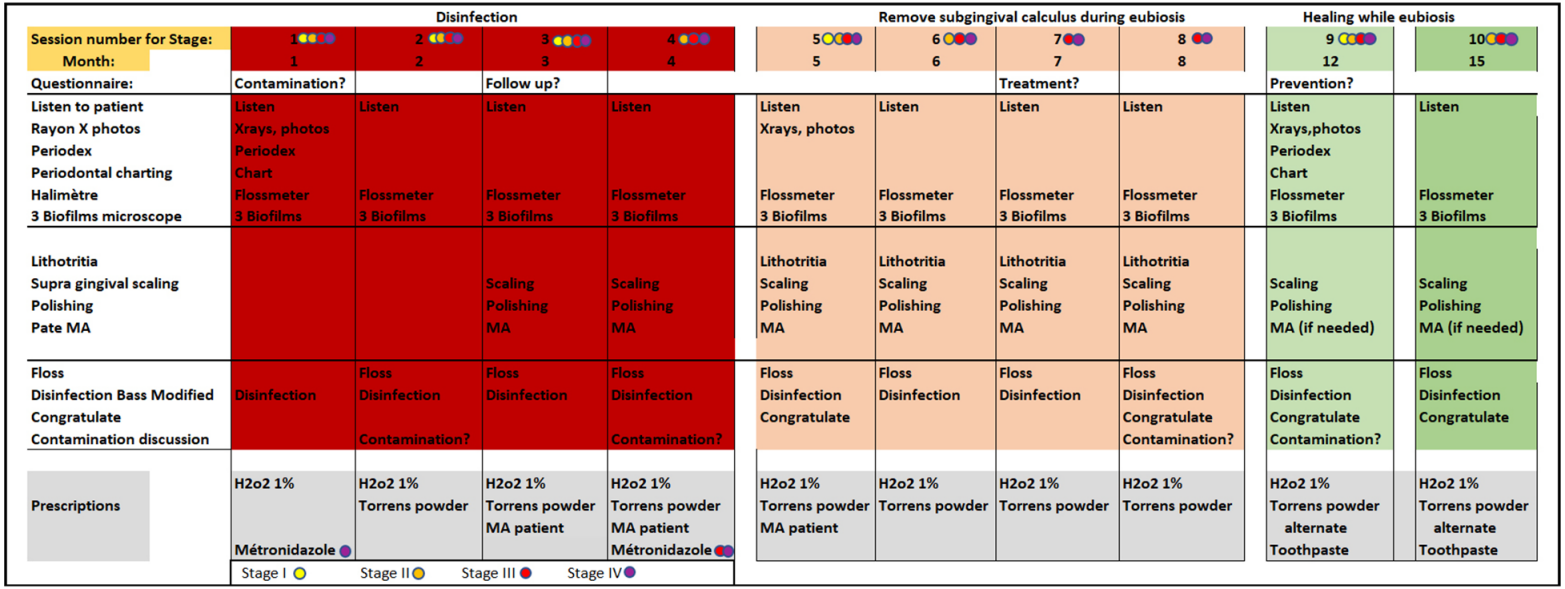
Detailed schedule of monthly therapy sessions as per PHPBD protocol. Practician activities during each visit are detailed. The grey areas at the bottom detail recommendations for disinfection as well as stage-dependent antiparasitic medication prescriptions. Colored circles allow the practitioner to adjust the treatment according to the different stages of the disease.

We systematically applied the PHPBD (see Data Availability Statement on Figshare.com) protocol on all patients as described below as well as in recent publication (18). A schematic view of the therapy is presented in Figure 1. The treatment aims at eliminating amoeba from the infected pockets, reducing lower motile bacterial activity below 1% as proposed by Savitt and Socransky (19) and confirm the absence of all inflammatory cells. Through successive microscopic clinical controls, we guide patients to reverse dysbiotic biofilm toward eubiosis and the absence of inflammatory cells. These biological markers were evaluated at each monthly appointment. Figure 2 provides a detailed description of procedures for each appointment at any stages.

The therapeutic goals for this approach are organized in three 4-month phases: first, restoring a normal commensal biofilm; second, removing subgingival calculi gently, using motorized ultrasonic or piezo devices instead of sharp instruments; finally, a healing phase to maintain hygiene control by the patient and ensure microscopic eubiosis. During the whole year of treatment, the patient is trained in clinic to use waxed floss and brush their teeth twice a day following the Bass modified 4-stroke method, as practiced during the third phase of each appointment. This brushing technique uses extemporaneously prepared 1% hydrogenated water instead of toothpaste. Then, a solution (Torrens Powder) of 6/7 sodium bicarbonate and 1/7 sodium chloride powder (wt/wt) as recommended by Lyons (11) is applied onto the patient's gum line using a wet clean finger, allowing to spit the excess solution. Hydrogen peroxide has been used in dentistry alone or in combination with salts for over 90 years (20). Deleterious effects of excess salt are produced on amoebae when added suddenly (21).

During the first phase of the therapy, a pharmacist preparation of 30 g 10% metronidazole cream mixed with 1,500,000 units nystatin and 2 ml pure anise oil for flavor is topically applied three times a day following the detection of amoebae (starting not earlier than the second visit) and until their disappearance. This mixture is also applied to the deepest pockets at the end of each appointment. If amoebae are initially present and persistent at month 4, they are then eliminated through systemic anti-parasitic medication (metronidazole, per os, 500 mg three times a day for 10–12 days). Exceptionally, this medication was prescribed earlier if it was judged that the patient risked losing a tooth. At this phase, no curettage nor root planning with sharp curettes is allowed, except for supragingival calculi removal instances.

**Figure 3:**
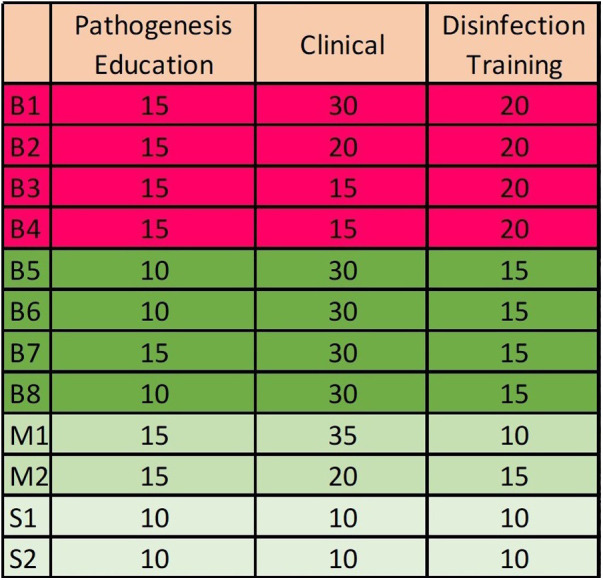
Timing allowed in minutes for each treatment sessions with clinician. B is used for “Bonner” clinic first to eighth appointment, M1 and M2 for maintenances and S1 and S2 for eventual subsequent needs.

During the second phase of the therapy, subgingival calculi are removed using ultrasonic and piezo tools only; sharp curettes are again not allowed. The most affected quadrant is done at the 5th appointment, while the others are treated during the three following monthly visits. At each appointment, general supragingival scaling and polishing are completed for all quadrants, while verifying the continued presence of a commensal biofilm at the three most affected teeth upon microscope examination. Adherence to hygiene protocols is practiced for the whole mouth, including dental floss, modified Bass technique and Torrens powder application. Adjustments and coaching are performed during the third part of each visit. Figure 3 details the domain of interest scheduled for each appointment.

During the third phase of the therapy, all four quadrants are considered cleaned, and 4 months of final healing is observed. At this stage, patients are allowed to use commercial toothpaste once a day, whereas the second brushing is done using 1% hydrogenated water and Torrens Powder application.

## 3 Results

### 3.1 Biofilm observations

**Figure 4:**
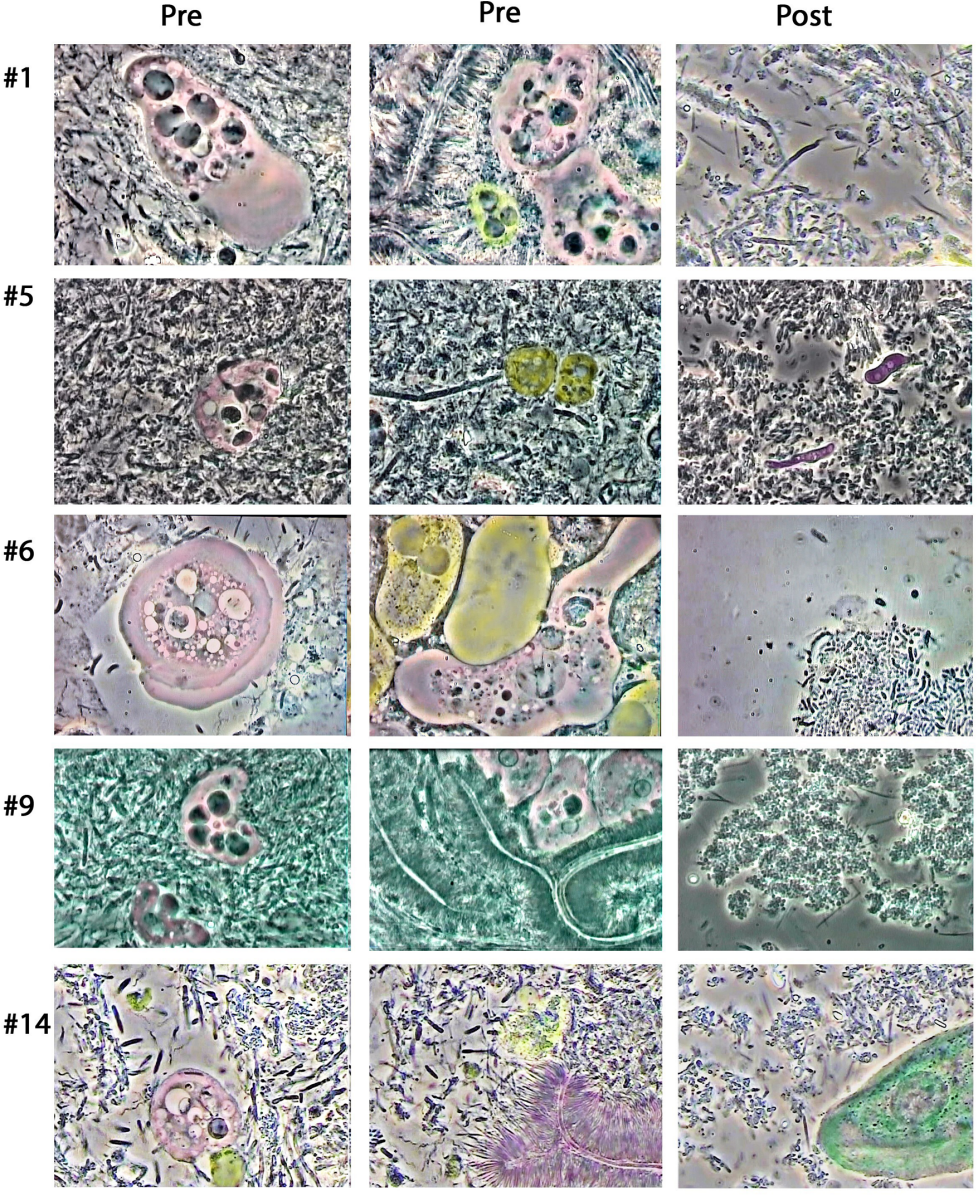
Representative microscopy images showing biofilms from five patients before (Pre) and post-treatment (Post). *E. gingivalis* amoebae are colorized in pink. Other identifiable cells were colorized in selected images, specifically neutrophils (yellow) and epithelial cells (green). Purple colorization was used for Candida yeast cells (Patient #5) as well as brush patterns (Patient #14). Two Trichomonas cells detected in patient #5 were colorized in dark yellow. The *E. gingivalis* cell in the first “Pre” image for patient #1 shows the presence of a ≈4 *μ*m round nucleus with karyosome, six darker inclusion vacuoles and a clear wide lobopode. The middle “Pre” image shows brush pattern formation surrounding two *E. gingivalis* amoebae and one neutrophil cell. For patient #5, the first left “Pre” image reveals an amoebae surrounded by hyper motile bacteria in the form of vibrios, bacilli and spirochetes, while completing exonucleophagy process. The second middle image shows the presence of two Trichomonas cells. For Patient #6, the left image shows a typical amoeba with lamellar pseudopod activity. The middle “Pre” image for this patient shows an amoeba in contact with two neutrophil cells, one with granules and a bilobed nucleus, while the middle one appears to have lost granules and nucleus [“ghost cell” (16)]. The left “Pre” image for patient #9 shows the presence of two amoebae, with the largest one containing five dark vacuoles typical of phagocytized neutrophil nuclei. The middle “Pre” picture shows brush patterns and three E. gingivalis on top. The left “Pre” image for patient #14 shows an amoeba, with contrasted nucleus, and what seems to be the remains of a neutrophil cell (yellow) as well as numerous highly motile bacilli, vibrios and spirochetes. The middle “Pre” image also shows remnants of a ruptured white blood cell (yellow), many motile bacteria and brush pattern association. Note that all five patients, “Post” images show eubiotic microbiota with non-motile cocci and filaments. Amoeba and white blood cells are not present. Similarly, no vibrio, bacilli or spirochetes are found. Two Candida cells are visible in the “Post” image from patient #5, and an epithelial cell is seen for patient #14.

*E. gingivalis* was identified by microscopy in all 20 patients; Patient *#*5 also bore *Trichomonas* parasites. Highly motile bacteria (spirochete species, vibrio and bacilli) as well as polymorphonuclear neutrophils (PMN) were detected in parasite smears from all patients, corroborating the loss of periodontal health and microscopic dysbiosis. Figure 4 shows two images per patient from five patients representative of this group with initial dysbiotic and inflammatory biofilm and final return to eubiosis without any visible inflammatory cells. Dysbiosis was evidenced mostly by the presence of active forms of *E. gingivalis*, *T. tenax*, very motile vibrios, bacilli and spirochetes. Brush pattern *Actinomyces* formation in close inquilinism with amoebae (16) was common among those patients. Neutrophils were occasionally found as “ghost cells” resulting from phagocytosis of the PMN nucleus by *E. gingivalis* through exonucleophagy, as described previously (14, 16). Final post-treatment microscopy images showed for all patients the presence of non-motile sparse bacteria in the form of cocci and filaments, some epithelial cells, and absence of inflammatory cells. Thus, all patients seemed to have reached microscopic eubiosis at the end of treatment. One patient (*#*5) had a small candida presence in the final biofilm. More extensive observations revealed that amoeba and other species representative of an infectious biofilm faded away between the first and fifth months of therapy. Macrophages tended to replace PMNs in the first phase of therapy but disappeared during the second phase (not shown). As the transmission mode of the parasite is still to be described, the different possible sources of reinfection were discussed and controlled during therapy: entourage, social interactions, spouses, close family, companion animals, and traveling in high-risk tropical contaminated water areas. Direct and indirect bacteria and parasite transmission possibilities were explained to the patients and thoroughly discussed.

### 3.2 Clinical observations

**Figure 5:**
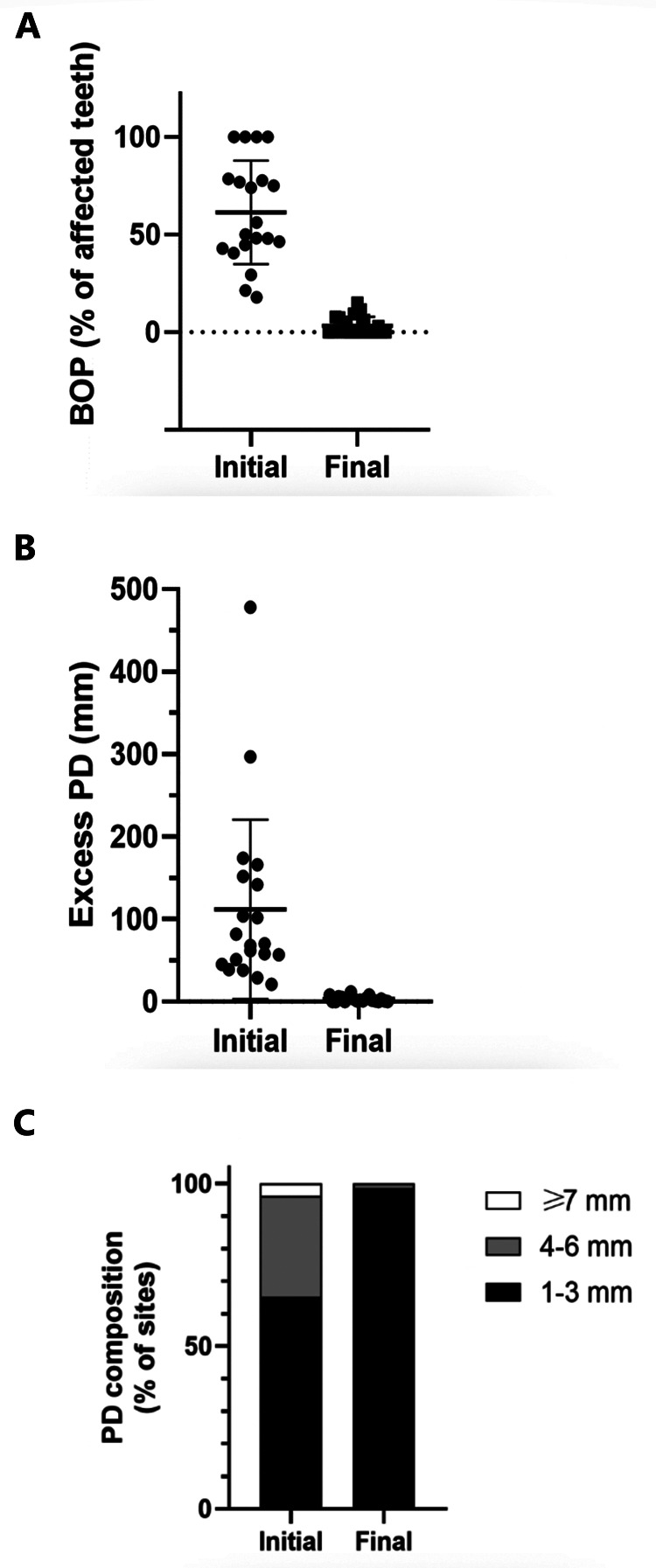
**(A)** Percentage of teeth presenting BOP per patient at initial and final visits. **(B)** Total PD in excess of 3 mm per patient at initial and final consultations (aggregated). **(C)** For each patient, total initial and final PD over 3 mm in 6 measurements for each tooth for all teeth.

**Figure 6:**
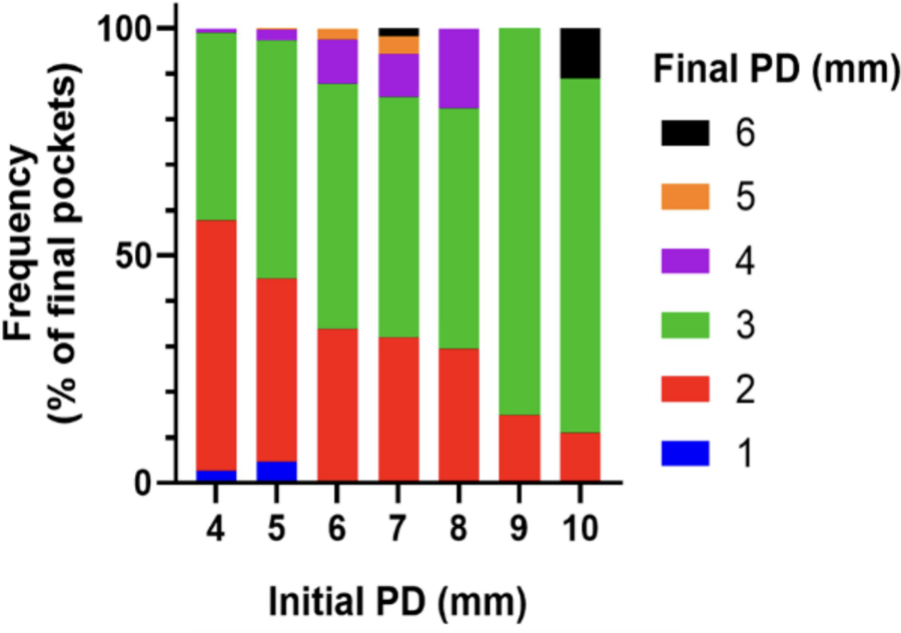
Pd size distribution at initial examination and final 12-month appointment.

**Figure 7:**
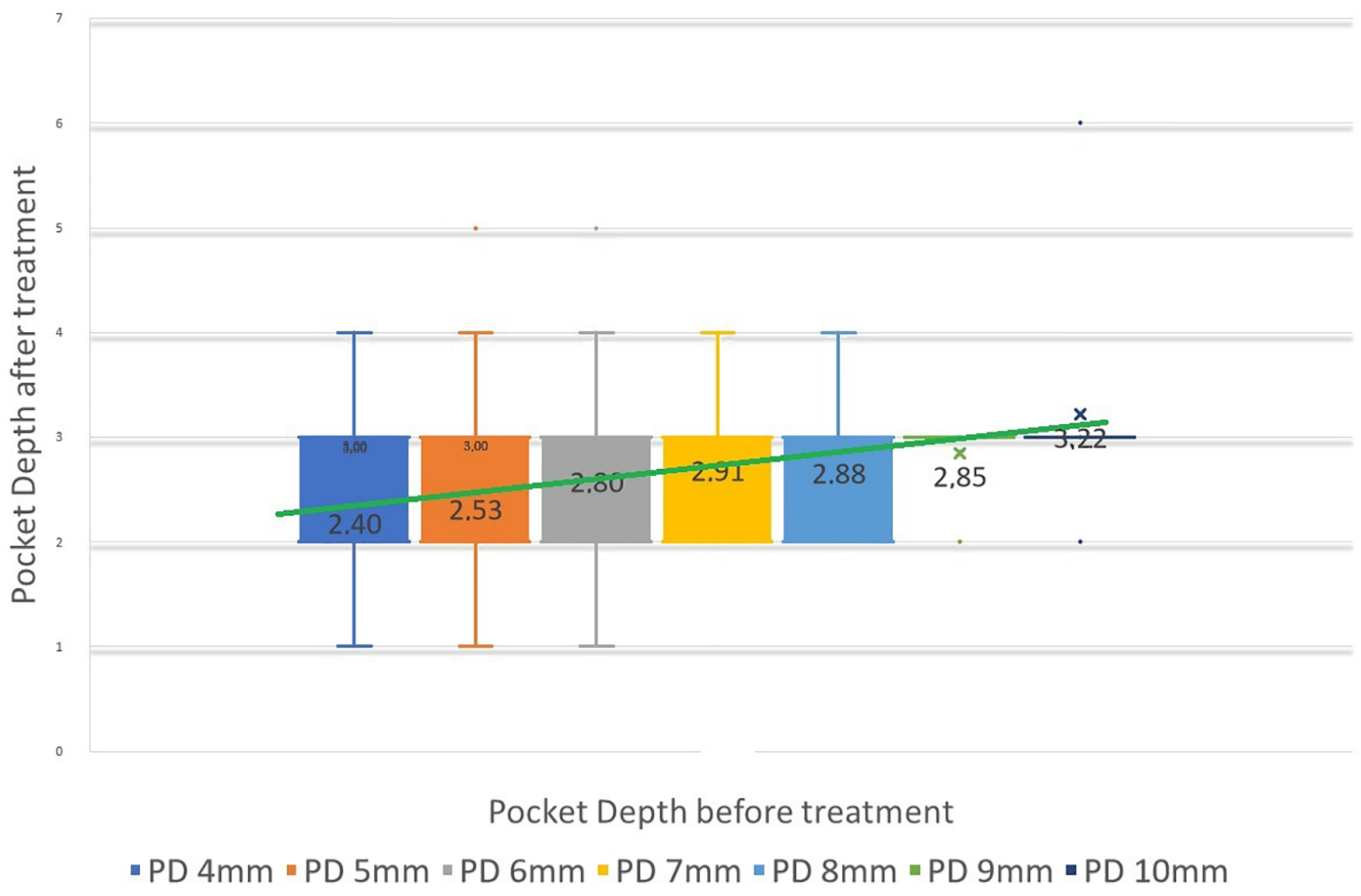
Median PD at final visit relative to initial PD height, for all PDs between 4 and 10 mm initially. Vertical bars represent 95% confidence interval. Green line can be seen as a predictive linear result of PD from initial pretreatment.

**Figure 8:**
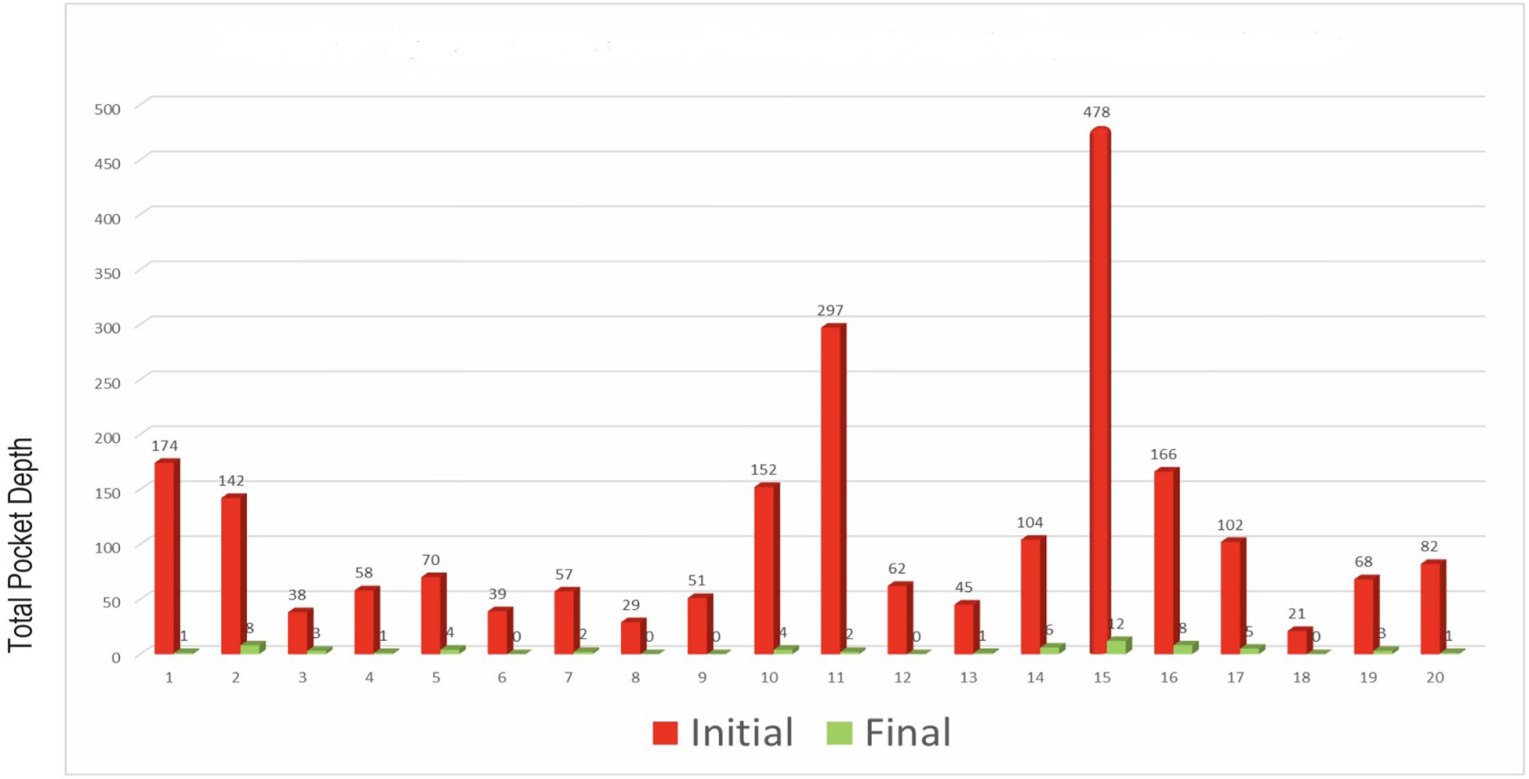
Total PD in excess of 3 mm for each 20 patients before and after treatment.

To quantitatively assess healing concomitant with microbiota rehabilitation, we measured BOP and PD throughout the therapy. We analyzed cumulative data per patient, total per group as well as the reduction between initial and final visits. As shown in Figure 5, all 20 patients presented with teeth that were bleeding upon probing (BOP-positive teeth); on average, 61% teeth were affected. In contrast, at the end of the therapy, most patients had no BOP-positive teeth (1.8% teeth on average) (Figure 5A final). As reported in Figure 5B, at the initial visit we determined that the total number of >3 mm PD per patient was quite elevated. At the final post-treatment visit, this number was found to be reduced to 3% on average. We also analyzed the size distribution of PDs (Figure 5C). At the first visit, nearly 35% of sites were diseased; specifically, 31% of the PDs analyzed were 4–6 mm in depth, and 3.8% were deeper than 7 mm. 65% of measured sites were not considered diseased, with a depth of 3 mm or less. At the end of the treatment, the total number of diseased pockets was greatly reduced compared with the first visit (96.5% and 100% reduction for the 4–6 mm and ≥7 mm groups respectively). Almost all measured PD (99%) were of less than 3 mm at end of treatment. The amoeba-specific PHPBD treatment reduced periodontal disease as judged by both BOP and PD markers. This was confirmed by examining the number of PDs over 3 mm for each patient (Figure 6) We also conducted an analysis of the fate of diseased pockets, by monitoring the final PD for each pocket. Results showed that all pockets underwent a significant reduction in size, regardless of their initial size. A more detailed analysis revealed that the median final depth of pockets ranged from 2.4 to 3.2 mm, depending on the initial size (Figure 7). Finally, we performed an analysis of cumulative excess PD for all pockets over 3 mm deep and for each patient, before and after each one therapy (Figure 8).

## 4 Discussion

Therapeutic management of a disease of unsure etiology is subtle. Several strategies can be undertaken for periodontitis; current ones rely on mechanical cleansing and promote the surgical removal of a portion of the oral tissues to, de facto, reduce pocket depth. Without the aid of microbial microscopic monitoring and inflammatory cells management, the initial result appears favorable; however, disregarding the infectious component of the disease yields a high failure rate. Some health practitioners targeted anaerobic organisms in the periodontal pockets and recommended disinfection [Popka (22), Lyons (11)]. Following this seminal path, we have integrated data from the literature and from unpublished observations into clinical care, leading us to propose the PHPBD protocol for therapeutic management of periodontitis. This protocol was successfully used in clinics, leading to 95.7% healing of periodontal pockets in 632 patients (6). In comparable non-surgical therapy, this number is usually around 18% for scaling and root planning only (23).

Standard non-surgical treatments for periodontitis generally lead to a modest reduction in pocket depth; in one study, for instance, 10-, 8- and 6-mm pockets are reduced after treatment to 6.7-, 5.5- and 4.4-mm pockets, respectively (24). These observations reflect an incomplete clinical resolution of sulcus healing regardless of the initial depth. In contrast, the antiparasitic method described in this study, employing strict microscopic control, leads to a greater reduction in PD, with a median final pocket depth between 2- to 3-mm for all periodontal pockets up to 9-mm deep at origin (Figure 7). This qualitative analysis allows us to predict the anticipated closure and healing of periodontal pockets. Specifically, pockets that are 10-, 9-, 8-, 7-, 6-, 5- and 4-mm deep will be reduced to 3.2-, 2.8-, 2.8-, 2.9-, 2.8-, 2.5- and 2.4-mm deep, respectively. In other words, the clinical result of pocket closure will be below or equal to the 3.22 mm bar for pockets up to almost 10-mm deep. This finding means that the outcome of the clinical data is predictable in most patients. We consider a PD measurement at or below 3 mm as a confirmation of clinical cure, especially if microscopy indicates microbiota representative of Socransky's green complex as detailed previously (18), the absence of any parasites and, importantly, the absence of inflammatory cells. Therefore, periodontal healing under these treatment conditions can be predicted and even announced to the patient if ready to fully participate in the process of this periodontal healing protocol.

Since the discovery of *E. gingivalis* in diseased mouth more than a century ago, the etiological link between this amoeba and periodontitis has clearly evolved toward it being a key inflammatory factor. Indeed, essential progress in the understanding of the pathophysiology of periodontitis was made by evidencing its correlation with the presence and aggressivity of *E. gingivalis* (14–16). Interestingly, the amoebae are never observed in apparently healthy sulci, but are readily detected as part of the flora specific to periodontitis. Whereas periodontal health correlates with microscopic observations of non-mobile cocci and bacilli (Figure 4), disease is characterized by a motile bacterial flora, presence of immune cells from the patient, and protozoa, mainly amoebae, and *T. tenax* promoting severe disease (25). This underlines the complex ecology of the periodontal pocket and the predictable parameters from health toward periodontitis. Altogether, current data suggest that presence of periodontitis requires its associated microbiota, and conversely, periodontitis-linked flora yields disease.

Microscopy-based analyses help improve our understanding of *E. gingivalis* parasitic behavior in periodontal disease. Images show the presence of denucleated neutrophils, sometimes described as “ghost” cells, as also observed in intestinal amebiasis dysentery (26–31). Removal of the insides, more specifically the nucleus, suggests the absence of normal NETosis. Neutrophil Extracellular Traps (NETs) are an organization of chromatin fibers exposed on the outside of the cell, and to which are attached enzymes, normally constituting lytic killing traps for pathogens (32). This phagocytosis of the nucleus by the amoeba (which we called “exonucleophagy”) (14), also described in our recent observational study (16) could contribute to the inability of the immune system to resolve the infection during periodontitis, leading to chronic inflammatory dysregulation. *E. gingivalis* has the potential to be considered as a hematophagous amoeba. Internal PMN enzymes may then be liberated and cause tissue destruction within the infected sulcus (33).

The adapted PHPBD protocol allows us to add biomarker status to the 2017 Staging and Grading World Workshop on Periodontitis (34), specifically eukaryotic microbes, bacterial cells, and inflammatory cells in the gingival pocket. *E. gingivalis* is present in various forms of periodontitis, including peri-implantitis (6), as an intrusive pathogen in light of its red blood cell phagocytosis capability, and of the degradation of host cellular immunity correlating with upregulated TNF*α* and IL8 as reported by Bao et al. (15). Its detection in periodontal sulcus should encourage clinicians to systematically propose antiparasitic therapy like those used in equivalent amoebaean medical diseases, thus ensuring better chances of success in promoting the return to an eubiotic flora free of inflammation. Some may counter that parasite correlation is not causation. At the very least, it is important to scientifically assess what could lead to an infectious response.

Let us recall here some Bradford Hill criteria that support causation:

1. Temporality: Parasites can establish themselves mainly on initial gingivitis and the number of parasites increases with the severity of the infection (16), if not concomitance with the age of the patient.
2. Strength: *E. gingivalis* exhibits high motility, leaving empty channels in the biofilm, manages to degranulate the surrounding WBCs, inserts its pseudopod inside the neutrophils and phagocytes their nuclei (exonucleophagy) through negative suction and peristaltic movement (16); *E. gingivalis* kills live epithelial cells (15); the amoeba reproduces by binary fission and can form pseudocysts under the action of the antimicrobial amoxycillin and metronidazole (35).
3. Consistency: Constant phagocytosis of red blood cells and white blood cells as a nourishing vacuole indicates a characteristic presence during periodontitis compared to health (16).
4. Plausibility: The attack of white blood cells leaves an inert neutrophil filled with enzymatic granules out of control on the surrounding tissues; the destruction of periodontal tissues may come from the undue breakdown of white blood cells induced by protozoa; *E. histolytica* and *E.* gingivalis possess the ability to cytolyze red cells and epithelial cells but *E. gingivalis* also cytolyzes leucocytes (11, 16, 36).
5. Coherence: Pathogenic characteristics typical of *E. gingivalis* due to capping phenomena (uroides 16), phagocytosis, damage to the immune capacity of the host and breakdown of epithelial cells (15).
6. Analogy: *E gingivalis* exhibits the same type of pathogenicity as *E. histolytica* without the need to resist stomach acidity. The periodontal pocket is reminiscent of “shirt button” ulcerations, i.e., lesions spreading “laterally” as the amoebae migrate parallel to the floor of the ulcer, strong nesting of certain regions of periodontal infection (15), anaerobic behavior and direct and indirect contamination (11).
7. Biological gradient: *E. gingivalis* infection correlates with the upregulation of the inflammatory cytokine IL8 (1,900-fold), the epithelial barrier gene MUC21 (8-fold), the collagenase protein MMP13 (11-fold); moreover, direct contact of *E. gingivalis* with gingival epithelial cells inhibits cell proliferation (15).

For all these reasons, rapid assertion such as Armitage (37) of an epiphenomenon can be described as purely dogmatic and though not fortuitous.

Active periodontitis presents as a specific parasitic infection superimposed on a gingivitis infection, which leads to destruction of the supporting tissues of the teeth, including connective tissue and bone. Clinically, periodontitis microbiota is relatively easy to monitor and reverse when using 100× and 1,000× hospital grade phase contrast microscope. Recurrence appears to be low if gingivitis is discarded as no white or red blood cells are observed in healthy gums. Gingival parasitic infection and inflammatory reaction are easily controlled via biofilm monitoring by microscopy as indicated by Keyes (38). Tissue regeneration seems to occur naturally when vertical bone defects are cleared of infection and inflammation (18).

It appears that parasitic infection in periodontal disease exploits the inflammatory response like the pathogenesis of liver amebiasis (28–30). Knowledge of the importance of non-bacterial microbial species in the pathogenesis of periodontitis may transform this disease from its status as a long-term, chronic disease to a short-term, transmissible, and curable one. It is coherent to claim that tissue destruction results from parasite-mediated inflammatory cells disruption, and trogocytosis (33). Disrupted PMNs likely lose their capacity to resolve infection through loss of apoptosis and normal NETose activity, and they may impede wound healing after exonucleophagy. This identifies, explains, and confirms the dysregulated immunity as the main contributor to periodontal disease from the parasitic infection (39). Necessary steps toward the resolution of inflammation include the elimination of pathogen parasites, the removal of motile bacteria and inflammatory cells, and the correction of local defects including calculus as well as control of gingivitis whether it is plaque-induced or not. It is interesting to note the recent use of topical statin drugs showing significant clinical results (40). Statins have antiparasitic activity, which may explain in part their effectiveness against periodontitis. Similarly, we propose that the long-recognized effectiveness of metronidazole as a periodontitis drug is linked to its antiparasitic activity rather than its light effects on the bacterial microbiome (41).

The absence of gingivitis should prevent further PMN-associated inflammatory responses and will limit parasitic infections. The management of parasitic transmission from the environment (via human oral transmissions, pets, infected water, dishes), whether direct or indirect, should be clearly encouraged. Based on parasitic and scrupulous biofilm monitoring, periodontal disease appears a relatively easy disease to regulate and heal.

This report contributes to defining the concept of periodontal health as the absence of pockets deeper than 3 mm, an inherent commensal biofilm or microscopic eubiosis consisting of non-motile cocci, filaments and some epithelial cells, the complete absence of parasites and granular white cell activity, the absence of bleeding, and environmental awareness. Complete periodontal cure appears to be a reachable goal through oral microbiota monitoring toward a fully healthy microbiota and elimination of inflammatory cells (42). From this study and previous ones, we conducted, therapeutic success seems to be reachable without the use of surgery, curettage, scaling and root planning with sharp curettes, but mainly by gentle subgingival calculus cleansing while clearing the sulcus of pathogenic microbes and inflammatory cells. Disinfection of the diseased sulcus should become the initial phase goal of treatment replacing scaling and root planning as a primary option. Active periodontitis also must be defined as a factual parasitosis superimposed on initial gingivitis induced by plaque, local factors, or eventual systemic factors.

This study points to the need of investigating the oral microbiota by different approaches including the scrupulous, repetitive use of microscopic phase contrast in periodontology, granulocyte counts and inventory of the entire microbiota, including the presence of parasites. Microscopy-based evidence leads us to propose that chronic periodontal disease is mostly a parasitic disease that is relatively easy to regulate and heal. Our understanding of the pathogenesis of periodontitis, as well as its treatment and prevention are likely to evolve from a chronic disease into a curable infection. Further research is needed to gather clinical and biological confirmation on a longer term and with a higher number of patients.

## Data Availability

The protocol, datasets generated and analyses in this study can be found in figshare.com Mark Bonner upload, https://doi.org/10.6084/m9.figshare.24585021.v1 and https://doi.org/10.6084/m9.figshare.25292917.v1.
